# Somatic mosaicism for copy-neutral loss of heterozygosity and DNA copy number variations in the human genome

**DOI:** 10.1186/s12864-015-1916-3

**Published:** 2015-09-16

**Authors:** Olga Žilina, Marina Koltšina, Raivo Raid, Ants Kurg, Neeme Tõnisson, Andres Salumets

**Affiliations:** Institute of Molecular and Cell Biology, University of Tartu, Riia 23, 51010 Tartu, Estonia; Department of Genetics, United Laboratories, Tartu University Hospital, L. Puusepa 2, 51014 Tartu, Estonia; Estonian Genome Center, University of Tartu, Riia 23b, 51010 Tartu, Estonia; Competence Centre on Health Technologies, Tiigi 61b, 50410 Tartu, Estonia; Department of Obstetrics and Gynaecology, University of Tartu, L. Puusepa 8, 51014 Tartu, Estonia; Institute of Bio- and Translational Medicine, University of Tartu, Ravila 19, 50411 Tartu, Estonia

**Keywords:** Array-CGH: array comparative genomic hybridization, Copy-neutral loss of heterozygosity (cn-LOH), Copy number variation (CNV), Human tissues, SNP genotyping arrays, Somatic mosaicism

## Abstract

**Background:**

Somatic mosaicism denotes the presence of genetically distinct populations of somatic cells in one individual who has developed from a single fertilised oocyte. Mosaicism may result from a mutation that occurs during postzygotic development and is propagated to only a subset of the adult cells. Our aim was to investigate both somatic mosaicism for copy-neutral loss of heterozygosity (cn-LOH) events and DNA copy number variations (CNVs) in fully differentiated tissues.

**Results:**

We studied panels of tissue samples (11–12 tissues per individual) from four autopsy subjects using high-resolution Illumina HumanOmniExpress-12 BeadChips to reveal the presence of possible intra-individual tissue-specific cn-LOH and CNV patterns.

We detected five mosaic cn-LOH regions >5 Mb in some tissue samples in three out of four individuals. We also detected three CNVs that affected only a portion of the tissues studied in one out of four individuals. These three somatic CNVs range from 123 to 796 kb and are also found in the general population. An attempt was made to explain the succession of genomic events that led to the observed somatic genetic mosaicism under the assumption that the specific mosaic patterns of CNV and cn-LOH changes reflect their formation during the postzygotic embryonic development of germinal layers and organ systems.

**Conclusions:**

Our results give further support to the idea that somatic mosaicism for CNVs, and also cn-LOHs, is a common phenomenon in phenotypically normal humans. Thus, the examination of only a single tissue might not provide enough information to diagnose potentially deleterious CNVs within an individual. During routine CNV and cn-LOH analysis, DNA derived from a buccal swab can be used in addition to blood DNA to get information about the CNV/cn-LOH content in tissues of both mesodermal and ectodermal origin. Currently, the real frequency and possible phenotypic consequences of both CNVs and cn-LOHs that display somatic mosaicism remain largely unknown. To answer these questions, future studies should involve larger cohorts of individuals and a range of tissues.

**Electronic supplementary material:**

The online version of this article (doi:10.1186/s12864-015-1916-3) contains supplementary material, which is available to authorized users.

## Background

Somatic mosaicism is defined as the presence of genetically distinct populations of somatic cells in one organism that has been derived from a single fertilised oocyte. Mosaicism may result from mutations of different scales that are propagated to only a subset of the adult cells during early development of the individual or later on during aging [1–4]. Generally, chromatid nondisjunction, erroneous DNA replication, faulty DNA repair mechanisms and recombination can lead to genetic alterations, such as aneuploidy, DNA copy number variations (CNVs), including deletions and duplications of chromosomal segments, or reciprocal loss and gain events that appear as copy-neutral loss of heterozygosity (cn-LOH) or acquired uniparental disomy (UPD) [5]. It is well known that somatic mosaicism for pathogenic mutations can result in miscarriages, congenital anomalies, developmental delay, and cancer [5–7]. However, benign mosaicism is common and has been reported to involve rearrangements of immunoglobulin and T-cell receptor genes in immune cells and mosaic aneuploidy in the human brain that is speculated to contribute to its functional diversity [1, 8–10]. In some cases, mosaicism does not have any phenotypic consequences and in others exhibits a diverse range of clinical phenotypes depending on the fraction of mosaicism within a given tissue. Due to this fact, but also because of the lack of appropriate methods and large datasets, until recently it was impossible to estimate accurately the frequency of mosaicism within the general population [5, 7]. However, in the recent meta-analysis involving 127,179 genotyped samples the detectable autosomal mosaicism was present in 0.73 % of individuals. It was shown that the number of mosaic events increases with age and that there is an inverse relationship between the rate of mosaicism and event size [11]. It should be mentioned though that the analysis was restricted to changes larger than 2 Mb in size and obviously the results represent the tip of the iceberg in relation to many smaller mosaic events. Furthermore, the structural genetic mosaicism was estimated based on either blood or buccal sample analysis, thus representing the mosaicism within a single tissue.

Early studies have focused on numerical chromosomal aneuploidy mosaicism, however, as techniques improved it was uncovered that somatic mosaicism for CNVs in normal human tissues is a rule rather than an exception [1, 2, 4, 12, 13]. In general, CNV is defined as a segment of DNA that is 1 kb or larger in size and is present in a variable copy number when compared with a reference human genome [14]. CNVs have been recognized as a key source of genetic variation among human individuals and occur in both phenotypically normal and affected subjects [14–16]. However, the phenotypic effects of CNVs are sometimes unclear and often depend on whether dosage-sensitive genes or regulatory sequences are affected by the genomic rearrangements [15].

The existence of somatic CNV mosaicism was first observed in a study of monozygotic (MZ) twins that displayed structural variations between them [12]. In total, 19 MZ twin pairs were screened for CNVs using a 32 K BAC array platform with a few cases displaying evidence of *de novo* somatic CNV events. This study thus refuted the common assumption that twins derived from the same zygote are genetically identical. These findings suggest that CNVs may arise from *de novo* events during early stages of embryogenesis, either before or just after the embryo has split into two individuals. However, only one type of tissue-blood-was used in this MZ twin study. On the contrary, a recent study indicated that large CNV discordance is rare between MZ twin pairs because only a single CNV difference was observed while genotyping 376 MZ twin pairs with Illumina Human610-Quad arrays [17].

Another study concentrated on the analysis of possible somatic CNV mosaicism in different tissues of the same individual. Panels of normal tissues from three males were studied using 32 K BAC arrays and at least six somatic CNVs that ranged from 82 to 176 kb were discovered in one or more tissues from the same subject [2]. These results suggested, for the first time, that somatic mosaicism for CNVs may be a common phenomenon.

Improvements in analysis technologies enabled one to study genetic alteration at the single-cell level and led to the discovery of mitotically derived genomic mosaicism, which is stable in different cell types within a single individual. Furthermore, once a CNV pattern has been established, the level of mosaicism seems to remain constant during the course of an entire lifetime [1, 18]. In addition, single-cell sequencing of human neurons revealed that 13 to 41 % of neuron cells have at least 1 Mb-scale *de novo* CNV, while a subset of these have highly aberrant genomes. Still, the functional meaning of neuron genome diversification remains to be determined [19].

Although almost every type of genetic variation has been implicated as a source of somatic variation including aneuploidy, CNVs, UPD, expansion of trinucleotide repeats, point mutations, mitotic recombination, translocation, and retrotransposition there are currently insufficient data on somatic mosaicism for cn-LOH events. Cn-LOHs can be defined as uninterrupted regions of homozygous alleles with genomic copy number state of 2. The minimal threshold for cn-LOH events varies across the studies and is usually set at 0.5–10 Mb [20]. In general, implication of SNP-based arrays enabled to study cn-LOH events, mainly in association with cancers because of their established role in carcinogenesis [21–24]. In addition, being helpful in detection of mosaicism for CNVs SNP arrays can also reveal mosaic cn-LOH events [7, 25]. In the meta-analysis conducted by Machiela and colleagues, approximately half of the events detected within blood or buccal tissue were mosaic cn-LOHs [11]. However, to the best of our knowledge, human tissue-level cn-LOH mosaicism has not been studied yet.

In view of the above the aim of our study was to investigate somatic mosaicism for cn-LOH events and CNVs in fully differentiated tissues. To accomplish this, we analysed DNA samples derived from 12 different tissues (not tested in previous studies) from four individuals using high-density HumanOmniExpress-12 BeadChips (Illumina, Inc; San Diego, CA, USA). This array platform allows one to detect DNA copy number changes, however, in contrast with BAC arrays, this also allows one to detect copy number neutral events. We assume that the somatic genetic mosaicism for CNVs and cn-LOHs found in our study reflects their formation during the postzygotic embryonic development of germinal layers and organ systems.

## Results

We employed Illumina HumanOmniExpress-12 BeadChip to detect DNA copy number changes and cn-LOH regions within 12 different tissues from three male (KA522, KT538, SJ600) and one female (BM419) autopsy patients (Additional file 1: Tables S1 and S2) and attempted to track their formation during the postzygotic embryonic development.

We identified 15 non-mosaic germ-line CNVs that were present in all tissues studied from a single individual. These included heterozygous deletions (*n* = 7; 46.7 %), heterozygous duplications (*n* = 7; 46.7 %), and a homozygous deletion (*n* = 1; 6.6 %). The total number of CNVs per individual ranged from 3 to 5 and the median and mean sizes of all CNV regions found were 62 and 78 kb, respectively (Additional file 1: Table S3).

**Fig. 1:**
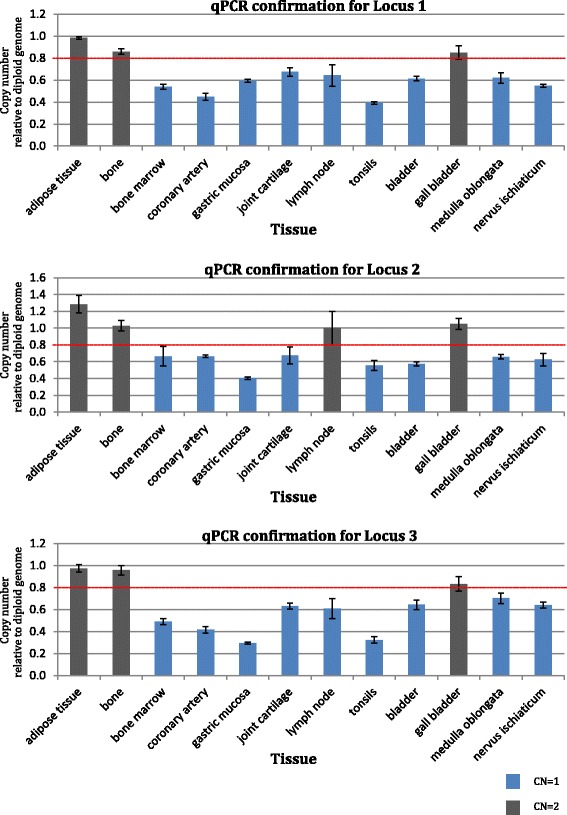
Validation of observed somatic CNVs using qPCR. The *bar charts* show haploid copy number for the 12 tissues studied. *Error bars* represent the standard error of the mean Ct difference between three technical replicates. Values *above* the *red line* indicate a normal diploid copy number (CN) of two, whereas those *below* the *red line* are indicative of a reduced diploid CN of one. Colour coding is applied to the respective copy numbers predicted by SNP-array: *black bars* represent tissues with CN = 2 and *blue bars* tissues with CN = 1 of the given CNV

In one of the four individuals studied (KT538), we detected three mosaic CNV regions in more than one tissue (Table 1 and Additional file 1: Table S4); while no somatic mosaicism for CNVs was observed in the other three individuals. The tissue-specific CNVs were validated using quantitative real-time PCR (qPCR) (Fig. 1) and found to be true positives.

**Table 1:** Three mosaic somatic CNV regions observed in one (KT538) individual (GRCh37/hg19)

Locus number	Chromosome	Start position	End position	Size (kb)	Copy number	Tissues	Genes
1	11	48,747,611	48,942,781	195–248	1	Bladder, bone marrow, coronary artery, gastric mucosa, ischiatic nerve, joint cartilage, lymph node, medulla oblongata, tonsils	None
					2	Adipose tissue, bone, gall bladder	
2	11	50,513,596	51,178,859	565–796	1	Bladder, bone marrow, coronary artery, gastric mucosa, ischiatic nerve, joint cartilage, medulla oblongata, tonsils	None
					2	Adipose tissue, bone, gall bladder, lymph node	
3	12	38,072,733	38,195,533	123–343	1	Bladder, bone marrow, coronary artery, gastric mucosa, ischiatic nerve, joint cartilage, lymph node, medulla oblongata, tonsils	None
					2	Adipose tissue, bone, gall bladder

The mosaic CNVs identified are located on chromosomes 11 and 12, and range from 123 to 796 kb in length. Two CNV regions (Locus 1 and Locus 3) display DNA copy number loss (CN = 1) in bladder, bone marrow, coronary artery, gastric mucosa, ischiatic nerve, joint cartilage, lymph node, medulla oblongata, and tonsils, while a diploid copy number (CN = 2) was observed in the three other tissues analysed: adipose tissue, bone, and gall bladder. Locus 2 displayed a DNA copy number loss (CN = 1) in bladder, bone marrow, coronary artery, gastric mucosa, ischiatic nerve, joint cartilage, medulla oblongata, and tonsils; and a diploid copy number (CN = 2) in adipose tissue, bone, gall bladder, and lymph node. Interestingly, differences in the length of the detected CNVs were observed between tissues (Table 1 and Additional file 1: Table S4). The size of Locus 1 ranged from 195 kb in bladder, bone marrow, coronary artery, ischiatic nerve, joint cartilage, lymph node, and medulla oblongata to 248 kb in gastric mucosa and tonsils. A similar tendency was observed within Locus 3 where the length of CNV ranged between 343 (gastric mucosa and tonsils) and 123 kb (in the seven other tissues analysed). Locus 2 displayed the length of 565 kb in bladder, ischiatic nerve, and medulla oblongata, 608 kb in bone marrow, coronary artery, and joint cartilage, and 790 and 796 kb in tonsils and gastric mucosa, respectively. It is noteworthy that the largest CNVs were observed in the same two tissues that contain endodermal components (gastric mucosa and tonsils) in all three regions. This suggests that these CNVs may have originated during early embryonic development.

The three mosaic CNVs detected were compared with those recurrently present in the Database of Genomic Variants (DGV) (http://dgv.tcag.ca/) and in the Estonian general population dataset (*n* = 6849), provided by the Estonian Genome Centre at University of Tartu [26]. All CNVs observed have been recurrently reported in the DGV and are also found in the Estonian general population (carrier frequencies of 0.4, 1.4, and 0.2 % in Loci 1, 2, and 3, respectively). When searching against the Ensembl 54 archive (May 2009) one of the CNV regions we detected (Locus 1) should contain the predicted protein-coding gene ENSG00000214907, while two other somatic CNV regions did not contain any known coding or regulatory elements. However, the ENSG00000214907 identifier is no longer present in the current Ensembl database (release 78, December 2014). In addition, we searched these CNV regions for regulatory elements using Ensembl regulation database (Homo Sapiens Regulatory Segments (GRCh37.p13)). There were no known regulatory elements in given regions.

In addition to CNVs, we also searched for the presence of cn-LOH events in all tissue samples. We set the minimum threshold for cn-LOH regions at 5 Mb. Altogether, we identified seven cn-LOH regions >5 Mb within tissues from four study subjects, five of which are mosaic and present in a subset of tissue samples from three (BM419, KT538 and KA522) out of four individuals (Table 2 and Additional file 1: Table S5). Two individuals (SJ600 and KA522) displayed overlapping non-mosaic cn-LOH regions on chromosome 9 (Table 2). The mosaic cn-LOH events were distributed on four different chromosomes (7, 8, 11, and X) and encompassed centromeres. Interestingly, the mosaic cn-LOH in chromosome 11 in individual KT538 overlapped with the mosaic CNVs in Locus 1 and 2, and was only present in tissues where CNV analysis revealed a diploid DNA copy number and not in the tissues with CN = 1 (Additional file 1: Tables S4 and S5). Thus, the monoallelic regions with reduced copy number (CN = 1) represent regions with deletions in some tissues, while in other tissues, the monoallelic regions without changes in the copy number (CN = 2) represent regions with cn-LOH.

**Table 2:** cn-LOH regions (>5 Mb) observed in different tissues from four individuals

Chromosome	Tissues
BM419	
Xp11.21-q11.1	Bone marrow, coronary artery, gall bladder
KT538	
8p11.21-q11.21	Adipose tissue, bone, gall bladder
11p11.2-q11	Adipose tissue, bone, gall bladder
KA522	
7p11.1-q11.21	Adipose tissue, bone, bone marrow, coronary artery, gall bladder, joint cartilage, lymph node,
9p12-p11.1	In all tissues studied
11p11.2-q11	Bone, gall bladder, medulla oblongata
SJ600	
9p13.1-p11.1	In all tissues studied

In order to determine if the CNV in Locus 3 overlaps with a cn-LOH that might have remained undetected using a threshold of 5 Mb, we reexamined this particular genomic region for smaller cn-LOH events, but did not find any.

## Discussion

To test the hypothesis that cells from different fully differentiated tissues could carry different CNV and cn-LOH patterns we investigated tissue panels (11–12 tissue samples per individual) from four individuals using high-density HumanOmniExpress-12 BeadChips that provide an effective resolution of 20 kb. High-resolution SNP arrays allow one to easily detect DNA copy number changes, however, unlike the array comparative genomic hybridization (array-CGH) technology used in previous studies [2, 12], the genotype information they provide also allows one to identify copy number neutral events such as cn-LOHs and UPD.

We detected three CNVs that affected only a portion of the tissues studied in one out of four individuals examined. No somatic mosaicism caused by CNVs was found in the other three individuals. The three identified genomic loci were present as a single copy (CN = 1) in the majority of the tissues studied and as a diploid copy (CN = 2) in the remaining tissues under study. Because the parental genomes were unavailable, we cannot conclusively prove if a single copy number or double copy number was the germ-line status and whether one copy was added or deleted during postzygotic development. A diploid copy number for the three genomic loci was detected in tissues of mesodermal origin (adipose tissue, bone, and lymph node) and one tissue that contains both endodermal and mesodermal components (gall bladder). A single copy number was present in tissues of different origins, such as mesodermal (joint cartilage and red bone marrow), endodermal (gastric mucosa and tonsils), ectodermal (coronary artery) and neuroectodermal (ischiatic nerve and medulla oblongata) derivatives, or those containing both endodermal and mesodermal components (bladder). Taking into account the specific sequence of the formation of germ layers during embryogenesis and the fact that the number of tissues with CN = 1 exceeded the number of tissues with CN = 2, we assume that the single copy was the original “zygotic state” in individual KT538 and an additional copy of a CNV region was added to a subset of the cells during mesodermal differentiation thereby leading to somatic mosaicism. However, these hypotheses are speculative and it is virtually impossible to predict either the time, cell types, or tissues where the mutation was introduced during early development.

Two out of the three observed CNVs did not overlap with any known genes or regulatory elements and most likely represent benign events. One observed CNV (Locus 1) was initially found to encompass a predicted protein-coding gene ENSG00000214907, however, this identifier has been removed from the newest version of the Ensembl database (release 78, December 2014). It has been suggested that CNVs that occur in a substantial fraction of normal cells might predispose these cells to specific disease-related phenotypes and lead to diseases, such as cancer, that derive from a few genetically altered cells [2, 27]. Still, phenotypic effects can only be expected if the reorganised genomic locus harbours some important genes or noncoding regulatory elements. Thus, the consequences of somatic mosaicism depend both on the number and type of the cells that are affected and also the genes and regulatory elements involved in the rearranged genomic locus [3]. It is notable that all mosaic CNVs found are reported recurrently in the DGV and are also present in the Estonian general population (the carrier frequency for Locus 2 is >1 %, but <1 % for Locus 1 and 3). This means that these CNVs may represent polymorphic variants and have a high probability of being benign. A similar tendency was found in a previous study of normal human tissues, where the majority of detected mosaic CNVs had previously been shown to be polymorphic [2].

The fact that our study revealed somatic CNV mosaicism in one (KT538) out of four studied individuals raises a number of questions. There is a high degree of probability that some CNVs remained undetected in our study due to the limitations of the array platform and analysis strategy used. Although HumanOmniExpress-12 BeadChips allow one to detect DNA copy number changes as small as ~20 kb, the smallest CNV found in our study was 42 kb in size and the overall number of CNVs detected was quite low. In order to minimize the number of false positive findings, only CNVs identified by two independent algorithms, QuantiSNP and PennCNV [28, 29], were marked for further study. This means that some true positive CNVs may have been missed and undetected mosaicism may be present in the other three individuals studied. Adoption of the newest methods for this kind of study would obviously provide more information. One option is the use of next generation sequencing (NGS) technologies to perform CNV analysis. Compared with array-based approaches that only enable one to examine genomic regions covered by predefined probes, short reads from NGS platforms are randomly sampled from the entire genome and provide single nucleotide resolution [30]. Furthermore, single-cell sequencing or parental-origin-determination fluorescence in situ hybridization (pod-FISH) were applied to map CNVs at the single-cell level [1, 19]. Moreover, it was recently demonstrated that some genomic loci, termed multiallelic CNVs (mCNVs), appear to be present in widely varying copy numbers in different human genomes [31, 32]. mCNVs are not routinely evaluated in array- or NGS-based genome-wide studies because these approaches have limitations in accurately measuring copy numbers greater than four. However, imputation from existing SNP data could be used to perform initial genome-wide scans to nominate specific mCNV loci for deeper analysis followed by direct molecular analysis [31]. Because mCNVs have only recently been recognized and our knowledge about them is limited, it cannot be excluded that this form of CNV may potentially display tissue-specific mosaicism.

In some cases, observed somatic CNV mosaicism can either be related to the variability in quality of DNA obtained from different tissue samples or to the accumulation of age-related somatic mutations [5, 33]. In our study, there were no deviations in the quality scores of tissue-specific DNA samples from KT538 (Additional file 1: Table S6). Furthermore, all the samples with a lower call rate in the SNP array analysis were excluded from subsequent bioinformatic analysis, which further reduces the possibility that the observed somatic mosaicism is caused by technological artefacts. To dispel any doubt we validated every observed mosaic CNVs by qPCR. The importance of qPCR replication was confirmed recently in a study of CNV concordance in 1097 MZ twin pairs, where most of the CNVs that were originally found to be discordant using Affymetrix 6.0 arrays and two CNV calling algorithms turned out to be present in both twins using qPCR validation procedures [34]. Mosaic CNVs detected in KT538 cannot be directly associated with aging-related accumulation of mutations, because this subject was only 40 years old (Additional file 1: Table S1). Moreover, a recent study of immortalized B-lymphoblastoid cells obtained before and after a 20 year interval from each of two subjects showed that the mosaic CNV patterns they acquired during early embryonic development remained stable throughout their lives [1]. However, another study, on the contrary, revealed a strong association between increased age and the number of detectable mosaic events >2 Mb, but at the same time it cannot be established whether the events were generated early in life or during aging [11].

In addition to CNVs, we identified five mosaic cn-LOH regions that encompass more than 5 Mb and these are present in only a subset of the tissues in three out of the four individuals studied. All detected cn-LOH events encompassed centromeres in contrast to the study of Machiela et al., who found the majority of mosaic copy-neutral events on the telomeric ends of chromosomes [11]. Although cn-LOH is the most common molecular genetic alteration observed in various human cancers, they are also commonly seen in healthy individuals [35, 36]. Regardless, knowledge regarding possible intra-individual cn-LOH mosaicism between different tissues is completely missing. To the best of our knowledge, this is the first study that reports somatic mosaicism for cn-LOH events on the level of different tissues of a single individual. It is most likely that the cn-LOH events we observed represent normal genomic variability because no malignant neoplastic diseases had been described in the medical histories of these three autopsy patients. Indeed, the detection of excessive homozygosity in and of itself does not allow for the diagnosis of any underlying condition and may be clinically benign [20, 35, 36]. Generally, regions with extended homozygosity can indicate ancestral homozygosity, uniparental disomy, or parental consanguinity. Short homozygous regions (up to 5 Mb) are considered ancestral markers and are present in all outbreed populations [37, 38].

We further analysed both the detected CNV and cn-LOH regions for possible overlap and it was found that in individual KT538 the mosaic cn-LOH in chromosome 11 overlaps with the mosaic CNVs in Locus 1 and 2, and is present only in tissues where CNV analysis revealed a diploid DNA copy number and not in the tissues with CN = 1 (Additional file 1: Tables S4 and S5). One can hypothesize that the single copy number was the “zygotic state” and another copy was added during subsequent embryonic development thereby generating the specific mosaic cn-LOH pattern we observe. However, this assumption is limited by the fact that in our study the size of cn-LOH region greatly exceeded the scale of CNV. In general, the mechanism for somatic cn-LOH mosaicism observed in this study remains unclear. Indeed, mitotic recombination followed by clonal expansion that could underlie copy-neutral telomeric events, can’t explain mosaic cn-LOHs encompassing centromeres. On the whole, breakpoint analysis of regions surrounding mosaic events might aid in understanding mechanisms responsible for event initiation, but SNP arrays do not provide sufficient probe density for this kind of analysis [11].

It appears that both cn-LOHs and CNVs can be implicated in somatic mosaicism which shows a great complexity and plasticity of the human genome. Somatic mosaicism should be considered as a form of intra-individual genetic variation, that may be implicated in somatic human diseases but also modify penetrance and/or expressivity of inherited disorders and late-onset multifactorial traits [25]. Although there were attempts to estimate the prevalence of somatic mosaicism in general population [11, 25], the real frequency should remain underestimated in these studies because of the established event size limits, the restrictions of the algorithms applied for the mosaicism detection and the limited number of different tissues analysed.

Most studies analyse DNA extracted from blood as one of the easily accessible sample materials. Nevertheless, it appears that there may be cases when blood alone does not fully represent the inherited germline alleles. It has been assumed that mosaicism for genomic alterations is much more likely to be observed in patients that display some clinical phenotype, however, it appears that tissue-specific genetic mosaicism also occurs in phenotypically normal individuals [2, 5–7, 12]. Indeed, our results agree with previous studies and suggest that some CNVs reported as germline ones may in fact represent post-zygotic somatic events. This, however, would not be observed in most studies that typically examine only one tissue and do not perform a full parental analysis for subjects under study [2, 12]. Therefore, studies that only use blood to perform CNV analysis should be taken with a certain degree of caution because the CNVs identified in the blood cells might not truly represent the genetic variability within other somatic and germline tissues. Thus, when possible, the use of a tissue panel is encouraged in CNV and cn-LOH studies to exclude potential misinterpretation caused by somatic mosaicism. However, it is typically not possible to obtain many types of tissues aside from the case when samples are taken from autopsy patients. Still, during routine analysis, DNA derived from a buccal swab can be used in addition to blood DNA to get information about the CNV and cn-LOH content in tissues of both mesodermal and ectodermal origin.

## Conclusions

Our results emphasize that somatic mosaicism for cn-LOHs and CNVs exists in normal human tissues and represents a common phenomenon that should be considered as a form of intra-individual genetic variation. Somatic chromosomal abnormalities may result from a mutation during postzygotic development which is propagated to only a subset of the adult cells. However, the phenotypic effect of somatic mosaicism depends on the nature of the mutation and the number and type of cells involved. Our data also indicate that the examination of only a single tissue is not enough to gain complete information about the CNV and cn-LOH content of the genome under study and the analysis of a tissue panel is warranted to obtain knowledge about possible variation in CNV and cn-LOH events across different tissues. However, both the real frequency and possible phenotypic consequences of both CNVs and cn-LOHs that display somatic mosaicism remain unknown and further studies involving larger sample cohorts are required to answer these questions.

## Methods

### Subjects and tissue samples

Four subjects of European ancestry were studied; one female (BM419) and three males (KA522, KT538 and SJ600). They were 60, 53, 40, and 54 years of age at time of death, respectively. Their causes of death were either cerebellar haemorrhage (BM419 and SJ600) or myocardial infarction with acute cardiovascular insufficiency (KA522 and KT538) and they were otherwise phenotypically normal. Between 4 and 8 h passed between the death of each subject and the collection of tissue samples (Additional file 1: Table S1). The Research Ethics Committee of the University of Tartu approved the collection of tissue samples for research (permission no 221/M-18). Written informed consent was obtained from next-of-kin to post-mortem individuals in order to collect the tissue panel during the autopsy and to publish individual patient data. The research was carried out according to the Helsinki Agreement.

In total, 12 tissue samples were collected from each individual: adipose tissue (subcutaneous), bladder, bone (hip joint), bone marrow (red), coronary artery, gall bladder, gastric mucosa, ischiatic nerve, joint cartilage, lymph node, medulla oblongata, and tonsils. The tissue panel for the female subject did not include joint cartilage (Additional file 1: Table S2). All samples were snap-frozen in liquid nitrogen and stored at −80 °C prior to analysis. The tissue samples were collected at the Pathology Centre within the North Estonia Medical Centre, Estonia and were examined and determined to be cancer-free by a pathologist.

The project was approved by the Research Ethics Committee from the University of Tartu.

### DNA extraction

Genomic DNA was extracted from 47 tissue samples using NucleoSpin® Tissue kit (Macherey-Nagel, Düren, Germany) according to the protocol provided by the manufacturer with minor modifications. To achieve a high yield and high concentration, DNA was eluted in 50 μl of a buffer preheated to 70 °C. The quality of the DNA samples was assessed and the concentration of DNA was measured using agarose gel electrophoresis together with a NanoDrop ND-1000 (Thermo Fisher Scientific, Inc, Wilmington, DE, USA) spectrophotometer (Additional file 1: Table S6).

### SNP arrays and data analysis

We studied both CNVs and cn-LOHs that display somatic mosaicism in various tissues from four unrelated subjects using HumanOmniExpress-12 BeadChip (Illumina, Inc, San Diego, CA, USA) arrays. These arrays contain 733,202 markers that cover the entire human genome with a median spacing of 2.1 kb and provide an average effective resolution of ~20 kb (i.e. 10 consecutive markers). We processed 200 ng of total DNA per sample and performed each assay according to the protocol supplied by the manufacturer.

The resulting array data was first analysed using Illumina GenomeStudio Software (Illumina, Inc) using a call rate of >99 % as the validity cutoff for each sample. With this criterion, three samples (SJ600-1; SJ600-2; SJ600-11) were excluded from further CNV analysis (Additional file 1: Table S2). In addition, relatedness analysis was performed using the PLINK software package (http://pngu.mgh.harvard.edu/purcell/plink/, [39]) to exclude the unlikely event of sample mix-ups between individuals.

We employed two independent algorithms, PennCNV and QuantiSNP, to detect DNA copy number changes using their default settings as previously described [28, 29]. For this, normalized signal intensities (log R ratios) and B-allele frequencies (BAF) for each marker were exported from GenomeStudio. Only CNVs detected by both programs and with a Log Bayes Factor >10 were included in further analysis. QuantiSNP was then used to identify cn-LOH regions for each tissue sample that passed the above criteria. The minimum threshold for each cn-LOH region was set at 5 Mb. All results were visually evaluated using the Illumina GenomeStudio software Genome Viewer tool (Illumina, Inc).

Next, we manually selected tissue-specific CNVs and cn-LOH regions that appeared only in a portion of tissues from the same individual. These CNVs were further compared with those recurrently present in both the DGV and in the Estonian general population (*n* = 6489, genotyped with HumanOmniExpress-12 BeadChips). The genomic context of each observed region was studied using Ensembl database (http://www.ensembl.org/) version 54 (based on NCBI36) and later re-evaluated using version 78 (based on GRCh38).

### Quantitative real-time PCR

Each CNV found to be mosaic was validated using qPCR on a 7900HT Fast Real-Time PCR System (Applied Biosystems, Foster City, CA, USA). One to two primer pairs per each CNV region were designed using the web-based service qRTDesigner 1.2 (http://bioinfo.ut.ee/gwRTqPCR/). Amplification mixtures (10 μl) contained 2× Maxima SYBR Green/ROX qPCR Master Mix (Thermo Fischer Scientific Inc., Vilnius, Lithuania), 250 nM forward and reverse primers and 2.5 ng gDNA. Three replicates were run for each targeted region using the following cycling conditions: 15 min at 95 °C, 40 cycles at 95 °C for 15 s and 60 °C for 60 s. After PCR amplification, a melting curve was generated to check the specificity of each PCR reaction (absence of primer dimers and other non-specific amplification products). To eliminate non-specific variations, such as differences in the amount of DNA input or presence of PCR inhibitors, C_t_ values were normalized using the C_t_ values of a reference region copy number of two. Data was acquired using SDS 2.4 software (Applied Biosystems) and further processed using a spreadsheet program. Relative quantification analysis was performed using the Pfaffl method while taking into account the amplification efficiencies of each primer pair [40].

## Additional file

Additional file 1: Table S1. General characteristics of the four subjects examined in the study. **Table S2.** Studied tissues and sample naming. **Table S3.** Summary of the non-mosaic (germ-line) CNV regions identified in all tissues of the body from four individuals studied. **Table S4.** Summary of tissue-specific CNVs observed in one of the four individuals studied (KT538). **Table S5.** Summary of tissue-specific cn-LOH events (>5 Mb) observed in three out of the four individuals studied. Table S6. DNA concentrations (ng/μl) and quality parameters (260/280 and 260/230 nm ratios) for each tissue type and subject. (PDF 548 kb)

## Abbreviations

BAF: B-allele frequency
CN: Copy number
cn-LOH: Copy-neutral loss of heterozygosity
CNV: Copy number variation
DGV: Database of Genomic Variants
mCNVs: Multiallelic copy number variations
MZ: Monozygotic
NGS: Next generation sequencing
qPCR: Quantitative polymerase chain reaction
SNP: Single nucleotide polymorphism
