# Supplementary material for: Somatic mosaicism for copy-neutral loss of heterozygosity and DNA copy number variations in the human genome
Source: BMC Genomics. 2015 Sep 16;16(1):703. doi: 10.1186/s12864-015-1916-3 (PMC4573927; doi:10.1186/s12864-015-1916-3)
Supplement: Additional file 1: Table S1. — General characteristics of the four subjects examined in the study. Table S2. Studied tissues and sample naming. Table S3. Summary of the non-mosaic (germ-line) CNV regions identified in all tissues of the body from four individuals studied. Table S4. Summary of tissue-specific CNVs observed in one of the four individuals studied (KT538). Table S5. Summary of tissue-specific cn-LOH events (>5 Mb) observed in three out of the four individuals studied. Table S6. DNA concentrations (ng/μl) and quality parameters (260/280 and 260/230 nm ratios) for each tissue type and subject. (PDF 548 kb) [file 12864_2015_1916_MOESM1_ESM.pdf]

Somatic mosaicism for DNA copy number variations and copy-neutral loss of heterozygosity in the human genome

Žilina O., Koltšina M., Metspalu A., Raid R., Kurg A., Tõnisson N., Salumets A.

Additional file

Table S1. General characteristics of the four subjects examined in the study.

**Subject ID**	**BM419**	**KA522**	**KT538**	**SJ600**
**Sex**	Female	Male	Male	Male
**Age (years)**	60	53	40	54
**Autolysis time (hours)**	4	8	8	8
**Clinical / pathological diagnosis**	cerebellar haemorrhage	acute cardiovascular insufficiency	myocardial infarction	cerebellar haemorrhage

Table S2. Studied tissues and sample naming.

Tissue	Sample ID	Subject 1 BM419	Subject 2 KA522	Subject 3 KT538	Subject 4 SJ600
Adipose tissue, subcutaneous	1	+	+	+	— *
Bladder	2	+	+	+	+
Bone, hip joint	3	+	+	+	— *
Bone marrow, red	4	+	+	+	+
Coronary artery	5	+	+	+	+
Gall Bladder	6	+	+	+	+
Gastric mucosa	7	+	+	+	+
Ischiatic nerve	8	+	+	+	+
Joint cartilage	9	—	+	+	+
Lymph node	10	+	+	+	+
Medulla oblongata	11	+	+	+	— *
Tonsils	12	+	+	+	+
**Total number of tissues studied:**		**11**	**12**	**12**	**9**

* Samples with a call rate <99% were excluded from further CNV analysis

Table S3. Summary of the non-mosaic (germ-line) CNV regions identified in all tissues of the body from four individuals studied. Chromosomal start and end positions of CNVs based on the GRCh37/hg19 human genome assembly.

Chromosome	Start Position (bp)	End Position (bp)	Length (kb)	No. of Probes	Copy Number
**KT538**					
10	47,543,322	47,703,869	160.5	50	3
11	55,365,761	55,427,700	61.9	19	0
12	63,942,649	64,118,558	175.9	19	3
**KA522**					
5	9,902,403	9,924,597	22.2	16	1
11	99,525,126	99,566,782	41.7	14	1
19	20,621,828	20,707,568	85.7	13	1
**SJ600**					
4	161,952,228	162,009,525	57.3	13	3
6	78,979,398	79,029,367	50	11	3
14	88,946,621	89,106,895	160.3	19	3
15	24,446,977	24,532,601	85.6	15	3
**BM419**					
3	65,196,123	65,214,685	18.6	11	1
5	97,048,466	97,099,320	50.9	18	1
7	157,734,314	157,799,412	65	28	3
19	6,958,134	7,018,869	60.7	24	1
19	43,390,362	43,465,171	74.8	11	1

Table S4. Summary of tissue-specific CNVs observed in one of the four individuals studied (KT538). Chromosomal start and end positions of CNVs based on the GRCh37/hg19 human genome assembly.

Sample Name	Chr.	Start Position (bp)	End Position (bp)	Length (kb)	No. of Probes	Copy Number	Max. Log BF (QuantiSNP)
**Locus 1**							
KT538_2	11	48,747,611	48,942,781	195.2	10	1	19.2
KT538_4	11	48,747,611	48,942,781	195.2	10	1	20.7
KT538_5	11	48,747,611	48,942,781	195.2	10	1	37.3
KT538_7	11	48,694,871	48,942,781	247.9	11	1	40.3
KT538_8	11	48,747,611	48,942,781	195.2	10	1	18.2
KT538_9	11	48,747,611	48,942,781	195.2	10	1	19.1
KT538_10	11	48,747,611	48,942,781	195.2	10	1	20.1
KT538_11	11	48,747,611	48,942,781	195.2	10	1	21.1
KT538_12	11	48,694,871	48,942,781	247.9	11	1	32.8
**Locus 2**							
KT538_2	11	50,513,596	51,178,859	565.3	18	1	27.1
KT538_4	11	50,513,596	51,222,017	608.4	21	1	34.6
KT538_5	11	50,513,596	51,222,017	608.4	21	1	32.5
KT538_7	11	50,325,741	51,222,017	796.3	36	1	108.4
KT538_8	11	50,513,596	51,178,859	565.3	18	1	16.2
KT538_9	11	50,513,596	51,222,017	608.4	21	1	26.7
KT538_11	11	50,513,596	51,178,859	565.3	18	1	30.9
KT538_12	11	50,332,064	51,222,017	789.9	35	1	84.1
**Locus 3**							
KT538_2	12	38,072,773	38,195,533	122.8	12	1	20.1
KT538_4	12	38,072,773	38,195,533	122.8	12	1	24.8
KT538_5	12	38,072,773	38,195,533	122.8	12	1	23.5
KT538_7	12	38,072,773	38,416,139	343.4	20	1	51.5
KT538_8	12	38,072,773	38,195,533	122.8	12	1	16.9
KT538_9	12	38,072,773	38,195,533	122.8	12	1	27.8
KT538_10	12	38,072,773	38,195,533	122.8	12	1	24.1
KT538_11	12	38,072,773	38,195,533	122.8	12	1	22.9
KT538_12	12	38,072,773	38,416,139	343.4	20	1	54.2

Chr.: chromosome

In the “Sample name” column, KT538 refers to the subject; numerals from 1 to 12 refer to the tissue sampled: 1 – Adipose tissue, 2 – Bladder, 3 – Bone, 4 – Bone marrow, 5 – Coronary artery, 6 – Gall bladder, 7 – Gastric mucosa, 8 – Ischiatic nerve, 9 – Joint cartilage, 10 – Lymph node, 11 – Medulla oblongata, 12 – Tonsils.

Table S5. Summary of tissue-specific cn-LOH events (>5Mb) observed in three out of the four individuals studied. Chromosomal start and end positions of cn-LOH based on the GRCh37/hg19 human genome assembly.

Sample Name	Chr.	Start Position (bp)	End Position (bp)	Length (Mb)	No. of Probes	Copy Number	Max. Log BF (QuantiSNP)
BM419_4	X	56,581,579	63,188,822	6.5	116	2	37.3
BM419_5	X	56,581,579	63,188,822	6.5	116	2	38.2
BM419_6	X	56,581,579	63,188,822	6.5	116	2	38.2
KT538_1	8	42,820,508	49,611,423	6.8	296	2	80.1
KT538_3	8	42,820,508	49,611,423	6.8	296	2	81.1
KT538_6	8	42,820,508	49,514,893	6.7	274	2	80.2
KT538_1	11	47,380,592	55,362,955	7.8	433	2	153.1
KT538_3	11	47,380,592	55,362,955	7.8	433	2	154.2
KT538_6	11	47,380,592	55,362,955	7.8	433	2	123.2
KA522_1	7	57,446,963	63,249,780	5.4	103	2	32.2
KA522_3	7	57,446,963	63,249,780	5.4	103	2	32.8
KA522_4	7	57,446,963	63,249,780	5.4	103	2	31.7
KA522_5	7	57,446,963	63,249,780	5.4	103	2	32.7
KA522_6	7	57,446,963	63,249,780	5.4	103	2	31
KA522_9	7	57,446,963	63,249,780	5.4	103	2	31.5
KA522_10	7	57,446,963	63,249,780	5.4	103	2	33.2
KA522_3	11	47,922,672	55,362,955	7.2	363	2	147.9
KA522_6	11	47,922,672	55,362,955	7.2	363	2	149.2
KA522_11	11	47,922,672	55,362,955	7.2	363	2	150.7

Chr.: chromosome
In the "Sample name" column, BM419, KT538, and KA522 refer to the subject; numerals from 1 to 12 refer to the tissue sampled: 1 – Adipose tissue, 2 – Bladder, 3 – Bone, 4 – Bone marrow, 5 – Coronary artery, 6 – Gall bladder, 7 – Gastric mucosa, 8 – Ischiatic nerve, 9 – Joint cartilage, 10 – Lymph node, 11 – Medulla oblongata, 12 – Tonsils.

Table S6. DNA concentrations (ng/μl) and quality parameters (260/280 and 260/230 nm ratios) for each tissue type and subject.

Tissue type	SJ600			KA522			KT538			BM419		
	ng/μl	260/280	260/230	ng/μl	260/280	260/230	ng/μl	260/280	260/230	ng/μl	260/280	260/230
Adipose tissue (subcutaneous)	92	1.9	2.2	55.2	1.7	1.7	73	1.9	1.8	55.2	1.9	2.2
Bladder	80	1.7	1.8	79.2	1.8	2.1	62.3	1.8	2.1	62.9	1.8	2.2
Bone, hip joint	75	1.9	2	70.2	1.6	1.4	67.3	2	2.2	62.7	1.8	1.8
Bone marrow (red)	145.1	1.8	2.3	192.4	1.9	2.3	75.9	1.8	2.0	84.1	1.8	2.1
Coronary artery	79.4	1.9	1.9	72.2	1.8	2.1	64.9	1.7	1.1	49.7	1.8	2.2
Gall bladder	61.6	1.8	2.2	97.3	1.9	2.3	81.7	1.9	2.3	68.3	1.9	2.2
Gastric mucosa	228.3	1.9	2.4	81.4	1.8	2.1	55.4	1.8	2.2	51.5	1.9	2.3
Joint cartilage	72.8	1.8	1.6	53.9	1.4	1.4	90	1.8	1.9	NA	NA	NA
Lymph node	220.4	1.8	2.4	98.2	1.8	2.0	91.3	1.9	2.4	57	1.7	2.1
Medulla oblongata	74.2	1.8	1.6	113.9	1.6	1.3	70	1.6	1.5	63.1	1.7	1.4
Nervus ischiaticus	59	1.8	1.9	67.2	1.9	2.1	36	1.7	1.3	51.9	1.8	1.3
Tonsils	87.1	1.8	2.2	284.5	1.9	2.4	94.7	1.8	2.1	255.8	1.9	2.4

NA - the tissue panel of the female subject BM419 did not include joint cartilage sample.
